# SIRT3 in Neural Stem Cells Attenuates Microglia Activation-Induced Oxidative Stress Injury Through Mitochondrial Pathway

**DOI:** 10.3389/fncel.2017.00007

**Published:** 2017-01-31

**Authors:** De-Qi Jiang, Yan Wang, Ming-Xing Li, Yan-Jiao Ma, Yong Wang

**Affiliations:** ^1^Department of Pharmacy, Zhujiang Hospital of Southern Medical UniversityGuangzhou, China; ^2^College of Biology and Pharmacy, Yulin Normal UniversityYulin, China

**Keywords:** neural stem cell, SIRT3, microglia activation, oxidative stress, cell apoptosis, ROS

## Abstract

Sirtuin 3 (SIRT3), a mitochondrial protein, is involved in energy metabolism, cell apoptosis and mitochondrial function. However, the role of SIRT3 in neural stem cells (NSCs) remains unknown. In previous studies, we found that microglia activation-induced cytotoxicity negatively regulated survival of NSCs, along with mitochondrial dysfunction. The aim of this study was to investigate the potential neuroprotective effects of SIRT3 on the microglia activation-induced oxidative stress injury in NSCs and its possible mechanisms. In the present study, microglia-NSCs co-culture system was used to demonstrate the crosstalk between both cell types. The cytotoxicity of microglia activation by Amyloid-β (Aβ) resulted in the accumulation of reactive oxygen species (ROS) and down-regulation of SIRT3, manganese superoxide dismutase (MnSOD) gene expression in NSCs, concomitant to cell cycle arrest at G_0_/G_1_ phase, increased cell apoptosis rate and opening of the mitochondrial permeability transition pore (mPTP) and enhanced mitochondrial membrane potential (ΔΨm) depolarization. Furthermore, SIRT3 knockdown in NSCs via small interfering RNA (siRNA) accelerated cell injury, whereas SIRT3 overexpression provided resistance to microglia activation-induced oxidative stress cellular damage. The mechanisms of SIRT3 attenuated activated microglia-induced NSC dysfunction included the decreased mPTP opening and cyclophilin D (CypD) protein expression, inhibition of mitochondrial cytochrome C (Cyt C) release to cytoplasm, declined Bax/B-cell lymphoma 2 (Bcl-2) ratio and reduced caspase-3/9 activity. Taken together, these data imply that SIRT3 ameliorates microglia activation-induced oxidative stress injury through mitochondrial apoptosis pathway in NSCs, these results may provide a novel intervention target for NSC survival.

## Introduction

The discovery of neural stem cell (NSC), especially adult NSC, brings new hope to neuronal regeneration and repair in the adult mammalian central nervous system. The process of neurogenesis continues throughout life, with thousands of new neurons generated every day in the mammalian brain (Aimone et al., 2010; Voloboueva and Giffard, 2011). NSC proliferation, apoptosis and other vital activities are regulated by complex signals in surrounding microenvironment (Sparkman and Johnson, 2008). Microenvironmental negative changes in neurodegenerative disorders and cognitive dysfunction play an important role in disturbed adult hippocampal neurogenesis (Drapeau and Nora Abrous, 2008; Li et al., 2008).

The neurodegenerative conditions are characterized by increased numbers of activated microglia and proinflammatory changes (Sparkman and Johnson, 2008; Voloboueva and Giffard, 2011). Activated microglia produce a variety of proinflammatory factors, including TNF-α, IL-6 and nitric oxide (NO), all of which are antineurogenic and oxidative stress induced factors (Voloboueva and Giffard, 2011). Reactive oxygen species (ROS)-induced inflammatory reaction destroys normal growth microenvironment of NSCs, which inhibits neuronal regeneration and mitochondrial functions (Liu et al., 2005; Torroglosa et al., 2007; Voloboueva and Giffard, 2011). High-level ROS also increases the concentration of intracellular Amyloid-β (Aβ) protein, leading to neuron damage (Leuner et al., 2012; Swerdlow et al., 2014). ROS facilitates cell death and proteinosis, which accelerate progression of neurodegenerative disorders, such as Alzheimer disease (AD; Leuner et al., 2012; Summers et al., 2014). Mitochondria are thought to play a critical role in neurogenesis. Neurons are especially sensitive and vulnerable to abnormality in mitochondrial function because of their high energy demand (Cui et al., 2012). Mitochondria protection and overexpression of anti-apoptosis protein improve cell viability and function of neural precursor cells *in vitro* (Chang et al., 2007; Doeppner et al., 2009; Voloboueva et al., 2010).

Sirtuin3 (SIRT3), a member of sirtuin gene family, is majorly located in mitochondria, showing histone deacetylase activity (Sundaresan et al., 2008). SIRT3 carries out various biological functions and is involved in regulation of oxidative stress, energy metabolism, protection of mitochondrial function and others, playing crucial roles in development and progression of neurodegenerative disorder, diabetes, cancer and other diseases (Zhou et al., 2014; Macconi et al., 2015; Quan et al., 2015; Yang et al., 2015). The expression of SIRT3 is also significantly down-regulated in cerebral cortex of AD rats (Yang et al., 2015). SIRT3 gene knockout increases neuron death caused by H_2_O_2_-induced oxidative stress, and aggravates degeneration of striatonigral dopaminergic neuron in PD rats (Liu et al., 2015). SIRT3 plays a certain role in the genesis and progression of nervous disorders. However, there is no evidence concerning the effects of SIRT3 in NSCs. Our previous study demonstrated that inflammatory damage, caused by microglia activation, induced cell apoptosis and mitochondrial injury in NSCs (Wang et al., 2015; Chen et al., 2016; Jiang et al., 2016).

The purpose of this study was to investigate change in expression of SIRT3 in NSCs upon exposure to microglia challenged with Amyloid-β (Aβ), and to explore whether SIRT3 exerts protection against microglia activation-induced cytotoxicity by alleviating oxidative stress injury and improving mitochondrial function. Furthermore, we also observed whether this neuroprotective effect of SIRT3 is regulated by a mechanism involving the mitochondrial survival pathway.

## Materials and Methods

### Cell Culture

The murine microglia cell line, BV-2, has been used previously as a substitute for primary microglia cells, as it exhibits very similar behavior. The C17.2 NSC line is capable of self-renewal and differentiation and has been used as a model system or cell therapy for neurodegenerative diseases (Wang et al., 2015). In this study, we used murine C17.2 cells line and BV-2 cells line as replacements for primary NSCs and microglia (MG), respectively. C17.2 cells were cultured at 37°C, 5% CO_2_ in high glucose DMEM (Gibco, Grand Island, NY, USA) supplemented with 10% (v/v) fetal bovine serum (Gibco, Grand Island, NY, USA), 5% (v/v) horse serum (Gibco, Grand Island, NY, USA) and 2 mM glutamine (Invitrogen, Carlsbad, CA, USA). BV-2 cells were propagated in flasks containing DMEM supplemented with 10% fetal bovine serum, at 37°C with 5% CO_2_.

### Transwell Co-Culture System

The microglia were cultured in transwell co-cultured system (Corning, NY, USA) that was placed above the NSC layer. Aβ peptide was added to a final concentration of 10 μM in microglia layer. The NSCs and microglia shared the same medium but no direct cell-cell interactions were possible due to the physical separation of the cells by a 0.4 μm polycarbonate membrane. We observed the response of the NSCs to the diffusible oxidative stress factors secreted by the stimulated microglia.

### Grouping

Experiments were performed on the following groups: (1) NSC: NSCs were cultured in the low chamber only for 48 h; (2) MG + NSC: NSCs were co-cultured with microglia for 48 h; (3) Aβ + NSC: NSCs were cultured in the low chamber with the addition of Aβ_1–42_ (10 μM, Sigma, San Francisco, CA, USA) for 48 h; (4) Aβ + MG + NSC (Control): NSCs were co-cultured with microglia with the addition of Aβ_1–42_ (10 μM) into the microglia chamber for 48 h, also known as the Control group in followed SIRT3 overexpression and interference tests; (5) Scrambled or siSIRT3: NSCs in the chamber were pretreated with negative control small interfering RNA (siRNA) or SIRT3 siRNA (Santa Cruz Biotechnology, Santa Cruz, CA, USA) for 6 h, respectively, then NSCs were co-cultured with microglia with the addition of Aβ_1–42_ (10 μM) into the microglia chamber for 48 h; and (6) Vector or SIRT3: NSCs in the chamber were pretreated with empty vector or SIRT3 expression plasmid (Vigenebio, Rockville, MD, USA) for 6 h, respectively, then NSCs were co-cultured with microglia with the addition of Aβ_1–42_ (10 μM) into the microglia chamber for 48 h.

### Measurement of Microglia-Derived Soluble Factors in Supernatant of Co-Culture System

The levels of TNF-α, IL-1β and IL-6 in the cell culture medium supernatants of co-culture system were detected with appropriate enzyme linked immunosorbent assay (ELISA) kits per manufacturer’s instructions. TNF-α kits was purchased from R&D System (Minneapolis, MN, USA), IL-1β and IL-6 kits were purchased from Genetimes Technology (Shanghai, China). The level of NO in supernatants was measured by Griess reagent. Griess reagent was obtained from Beyotime Biotechnology (Shanghai, China). The concentration of soluble cytokines in each sample was determined from a standard curve generated by using positive controls provided in the kits.

### Transient Transfection with the SIRT3 Expression Plasmid and siRNA

The recombinant adenoviral vector overexpressing SIRT3 (SIRT3 group) and the empty vector (Vector group) were produced by Vigenebio (Rockville, MD, USA). When the NSCs reached 50% confluence, recombinant adenovirus was added at a multiplicity of infection of 100 for 6 h. The SIRT3 and manganese superoxide dismutase (MnSOD) genes expression in NSCs were evaluated by quantitative reverse transcription-polymerase chain reaction (qRT-PCR) and western blotting. Negative control siRNA (Scrambled group) and SIRT3 siRNA (siSIRT3 group) were obtained from Santa Cruz Biotechnology (Santa Cruz, CA, USA). NSCs were transiently transfected using Lipofectamine 2000 (Invitrogen, Carlsbad, CA, USA) according to the manufacturer’s instructions.

### Evaluation of ROS Production

The fluorescent probe dihydroethidium (DHE; Beyotime Biotechnology, Shanghai, China) and hydrogen peroxide assay kit (Beyotime Biotechnology, Shanghai, China) were used to evaluate the intracellular accumulation of superoxide anion and hydrogen peroxide according to the manufacturer’s recommendations, respectively. Briefly, experiments were performed in 24-wells in which cells were incubated with 5 μM DHE for 30 min at 37°C, or hydrogen peroxide reaction solution for 30 min at room temperature. Cells were subsequently washed with PBS, lysed and transferred to a 96-well black plate for measurement of fluorescence intensity with a plate reader (Thermo Fisher Scientific, Waltham, MA, USA).

### Quantitative Reverse Transcription-Polymerase Chain Reaction (qRT-PCR)

Total RNA was isolated with TRIzol regent (Life Technology, Carlsbad, CA, USA). The expression of mRNA level was analyzed by qRT-PCR analysis as described previously (Wang et al., 2015). Specific primers were synthesized by Sangon Biotech (Shanghai, China), and the sequences were described as follows:

- SIRT3: forward: 5’-ATGCCTGAAGACAGCTCCAACAC-3’,
- reverse: 5’-AGACATCCCTGGTCAGCCTTTCC-3’.
- MnSOD: forward: 5’-TAAGGAGAAGCTGACAGCCGTGT-3’,
- reverse: 5’-AGAGCAGGCAGCAATCTGTAAGC-3’.

### Western Blotting Analysis

Western blotting was performed as previously described (Wang et al., 2015). Briefly, cell protein extracts (15–20 μg) were separated by 10–15% SDS-PAGE and transferred to PVDF membranes. Proteins were detected with the following antibodies: SIRT3 (1:1000, Cell Signaling Technology, Boston, MA, USA), MnSOD (1:1000, Proteintech, Chicago, IL, USA), cyclophilin D (CypD; 1:1000, Abcam, Cambridge, UK), cytochrome C (Cyt C; 1:1000, Cell Signaling Technology), cleaved caspase-3 (1:1000, Cell Signaling Technology), Bax (1:1000, Cell Signaling Technology, Boston, MA, USA), B-cell lymphoma 2 (Bcl-2; 1:1000, Cell Signaling Technology, Boston, MA, USA), COX-IV (1:1000, Abcam, Cambridge, MA, USA) and GAPDH (1:2000, Proteintech, Chicago, IL, USA).

### Cell Apoptosis and Cell Cycle Analysis

Cell apoptosis of NSCs was analyzed with FACSCalibur flow cytometer (BD, USA) using Annexin-V/propidium iodide (PI) assays according to the manufacturer’s instructions (BD Biosciences, Franklin Lakes, NJ, USA) as described previously (Wang et al., 2015). The NSCs were harvested, washed and then fixed with 70% ethanol. The cell fixation took overnight time in −20°C freezer. The next day, the fixed cells were centrifuged to collect pellet, washed with ice-cold PBS and resuspended with staining buffer containing 50 mg/mL PI, 0.1% Triton X-100, 0.1% sodium citrate and 100 mg/mL RNase. The cell suspension was incubated in the dark condition for 30 min at room temperature. The stained cells were then analyzed with flow cytometer.

### Measurement of the Mitochondrial Membrane Potential (ΔΨm)

The NSCs were incubated with JC-1 (Beyotime Biotechnology, Shanghai, China) at 37°C for 20 min and were then washed 2× with the dyeing buffer. The fluorescence level was measured immediately at a single excitation wavelength (488 nm) and dual emission wavelengths (shift from 530 nm to 590 nm) using a high-content screening platform (Thermo Fisher Scientific, Waltham, MA, USA).

### Mitochondrial Permeability Transition Pore (mPTP) Opening Assay

NSCs were loaded with calcein-AM (Santa Cruz Biotechnology, Santa Cruz, CA, USA) and CoCl_2_ (Sigma-Aldrich, St Louis, MO, USA), incubated for 15 min at 37°C. Cells were centrifuged, washed, and cell pellets were resuspended in 0.4 ml PBS and analyzed for mitochondrial calcein-AM fluorescence by flow cytometry analysis. The excitation/emission fluorescence for calcein-AM is 494/517 nm. There is an inverse relationship between calcein-AM fluorescence intensity and the number of mitochondrial permeability transition pore (mPTP) opening.

### Detection of Caspase-3 and Caspase-9 Enzyme Activities

Caspase-3 and caspase-9 enzyme activities were assayed by the spectrophotometric method according to the manufacturer’s instructions, the caspase-3 and caspase-9 assay kits were obtained from Beyotime (Shanghai, China).

### Statistical Analysis

Data were expressed as mean ± standard deviation and analyzed using SPSS version 20.0 (IBM, Armonk, NY, USA). The Kruskal-Wallis analysis followed by Dunn-Bonferroni *post hoc* comparisons were used to compare the difference in multiple groups. Values were considered significant at *p* < 0.05.

## Results

### Microglia Activation Induces Oxidative Stress Injury and Decreases SIRT3, MnSOD Expression in NSCs

Our previous studies demonstrated that microglia activation-derived inflammatory cytokines could effectively inhibit proliferation and promote apoptosis of NSCs (Wang et al., 2015; Jiang et al., 2016). We want to see whether microglia activation brings about oxidative stress injury to NSCs. The effects of microglia activation on the intracellular ROS production, cell apoptosis, cell cycle distribution, mitochondrial ΔΨm and the opening of mPTP in NSCs were explored.

**Figure 1:**
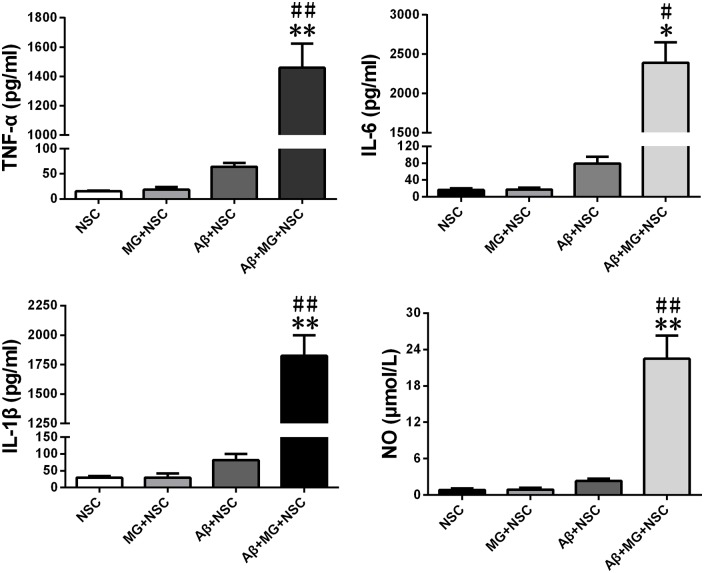
**The levels of nitric oxide (NO), TNF-α, IL-1β and IL-6 in supernatant of co-culture system are increased.** After microglia-neural stem cells (NSCs) co-cultured for 48 h, the concentration of TNF-α, IL-1β, IL-6 and NO in cell culture medium supernatant were measured by enzyme linked immunosorbent assay (ELISA) or Griess reagent. Data are presented as mean ± S.D. (*n* = 4). *P < 0.05, **P < 0.01 vs. the MG + NSC group. ^#^*P* < 0.05, ^##^*P* < 0.01 vs. the Amyloid-β (Aβ) + NSC group, respectively.

**Figure 2:**
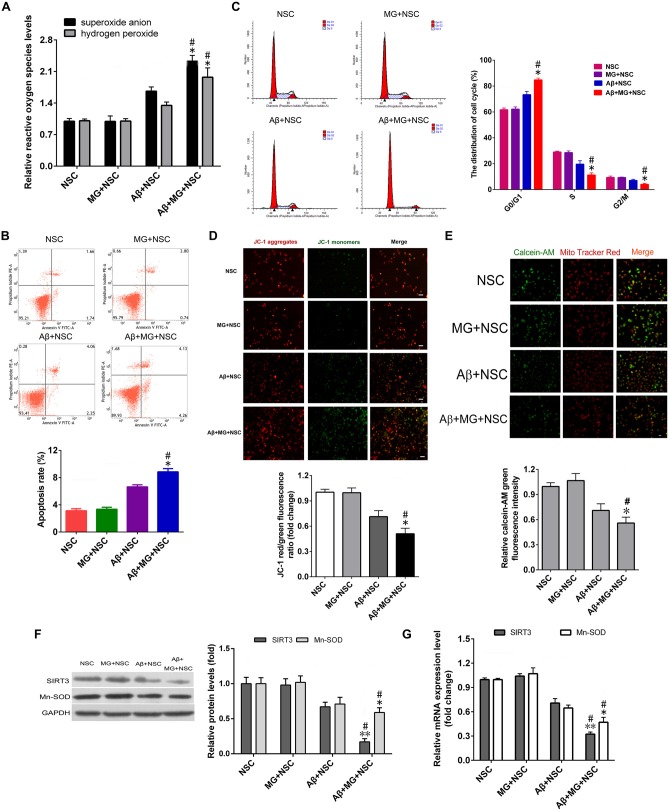
**Microglia activation induces oxidative stress injury and decreased sirtuin 3 (SIRT3), manganese superoxide dismutase (MnSOD) expression in NSCs. (A)** The relative changes of superoxide anion and hydrogen peroxide levels in NSCs treated with or without 10 μM Aβ, co-cultured with or without microglia (MG) were evaluated by fluorescent probe dihydroethidium (DHE) and hydrogen peroxide assay kit, respectively. **(B)** Representative images (up panel) and quantification (down panel) of cell apoptosis in NSCs were determined by Annexin-V/propidium iodide (PI) assay. **(C)** Representative images (left panel) and quantification (right panel) of cell cycle distribution in NSCs was detected by flow cytometry. **(D)** Representative images (up panel) and quantification (down panel) of mitochondrial depolarization were assessed as the fluorescence shift of JC-1 from red to green by high-content screening platform analysis, *Scale bar* = 100 μm. **(E)** Representative images (up panel) and quantification (down panel) of the NSCs loaded with calcein AM (green) and CoCl_2_ (cytosolic calcein quencher) to determine the calcein fluorescence in the mitochondria were analyzed by flow cytometry analysis, MitoTracker (red) staining was utilized for localization of mitochondria, *Scale bar* = 50 μm. The expression of SIRT3, MnSOD in NSC, MG + NSC, Aβ + NSC and Aβ + MG + NSC group was analyzed by Western blot **(F)** and quantitative reverse transcription-polymerase chain reaction (qRT-PCR; **G**). Results in **(A,D–G)** are normalized to that of the NSC group. Data are presented as mean ± S.D. (*n* = 3). *P < 0.05, **P < 0.01 vs. the MG + NSC group. ^#^*P* < 0.05 vs. the Aβ + NSC group.

The levels of NO, TNF-α, IL-6 and IL-1β in cell culture medium supernatant of co-culture system were all significantly elevated after the addition of Aβ for 48 h (Figure 1). Results of assays using the fluorescent probes DHE and hydrogen peroxide reagent kit showed that microglia activation remarkably elevated the intracellular ROS levels in NSCs (Figure 2A). The results of the annexin V-FITC/PI double-staining assays indicated that microglia activation dramatically increased the apoptosis rate of NSCs (Figure 2B). As shown in Figure 2C, microglia activation resulted in the ascending proportion of G_0_/G_1_ phase cells significantly, and the proportion of S and G_2_/M phase cells descended markedly, which tested by flow cytometry. Assays using the fluorescent probes JC-1 and calcein-AM verified that microglia activation promoted ΔΨm depolarization (Figure 2D) and mPTP opening in NSCs (Figure 2E).

To determine the effects of microglia activation-induced oxidative stress on the SIRT3 and MnSOD expression in NSCs. MnSOD, a downstream target gene of SIRT3, is a critical antioxidant enzyme that eliminate ROS in mitochondria. Western blotting and qRT-PCR assays were performed to assess the SIRT3 and MnSOD gene expression. Figures 2F,G displayed that microglia activation made a significant reduction of SIRT3 and MnSOD expression in both mRNA and protein levels. These results elucidated that microglia activation resulted in oxidative stress injury, mitochondrial dysfunction and decreased expression of SIRT3 and MnSOD in NSCs.

### Aggravation of Microglia Activation-Induced Cytotoxicity by SIRT3-siRNA Transfection

**Figure 3:**
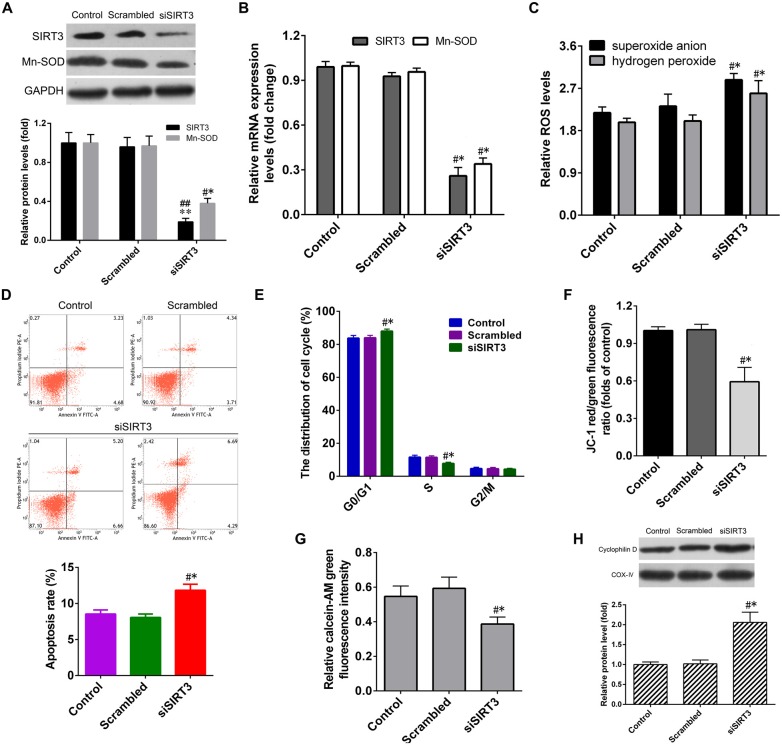
**SIRT3 knockdown by small interfering RNA (siRNA) transfection exacerbates cell dysfunction in NSCs. (A)** Representative western blot (up panel) and densitometric quantification (down panel) of SIRT3, MnSOD protein expression in NSCs treated with scrambled siRNA (Scrambled group) or SIRT3 siRNA (siSIRT3 group) for 48 h. **(B)** The mRNA expression of SIRT3, MnSOD in NSCs treated with scrambled RNA or SIRT3 siRNA for 36 h. **(C)** The levels of superoxide anion and hydrogen peroxide in NSCs were detected. The cell apoptosis rate **(D)**, cell cycle distribution **(E)** and mitochondrial depolarization **(F)** in the Control, Scrambled and siSIRT3 groups were analyzed by methods as showed in Figure 2, respectively. **(G)** The mitochondrial permeability transition pore (mPTP) opening infected with SIRT3 siRNA for 48 h was determined according to calcein-AM fluorescence intensity by flow cytometry. **(H)** Representative western blot (up panel) and densitometric quantification (down panel) of cyclophilin D protein expression in NSCs treated with scrambled RNA or SIRT3 siRNA for 48 h, COX-IV was used as mitochondrial markers. Results in **(A–C,F–H)** are expressed as fold change over that of Control group. Data are obtained in NSCs co-cultured with microglia challenged with 10 μM Aβ exposure. Data are presented as mean ± S.D. (*n* = 3). *P < 0.05, **P < 0.01 vs. the Control group. ^#^*P* < 0.05, ^##^*P* < 0.01 vs. the Scrambled group.

To evaluate whether SIRT3 knockdown in NSCs deteriorates microglia activation-induced cytotoxicity. After transfection with the SIRT3-siRNA (siSIRT3 group), the SIRT3 and MnSOD mRNA levels in NSCs significantly dropped by about 75% compared to that in untransfected cells (control group) or in cells transfected with the negative siRNA (scrambled group), and the SIRT3 and MnSOD protein levels in NSCs of SIRT3 group were also lower than that in control or scrambled group (Figures 3A,B).

The results depicted in Figure 3C manifested that SIRT3 knockdown further accelerated microglia activation-induced ROS production. The rate of NSCs apoptosis in siSIRT3 group was much higher than that in the control or scrambled group (Figure 3D). SIRT3 knockdown further increased the proportion of G_0_/G_1_ phase cells significantly, while S phase cells proportion declined dramatically compared to that in the control or scrambled group (Figure 3E). Assays using the fluorescent probes JC-1 and calcein-AM illuminated that SIRT3 knockdown promoted microglia activation-induced ΔΨm depolarization (Figure 3F) and mPTP opening (Figure 3G) in NSCs, consistent with enhanced levels of CypD protein expression (Figure 3H). In summary, these findings provided important evidence that inhibition of SIRT3 expression exacerbated microglia activation-induced cell damage in NSCs.

### SIRT3 Overexpression Protects Against Microglia Activation-Induced Cytotoxicity

**Figure 4:**
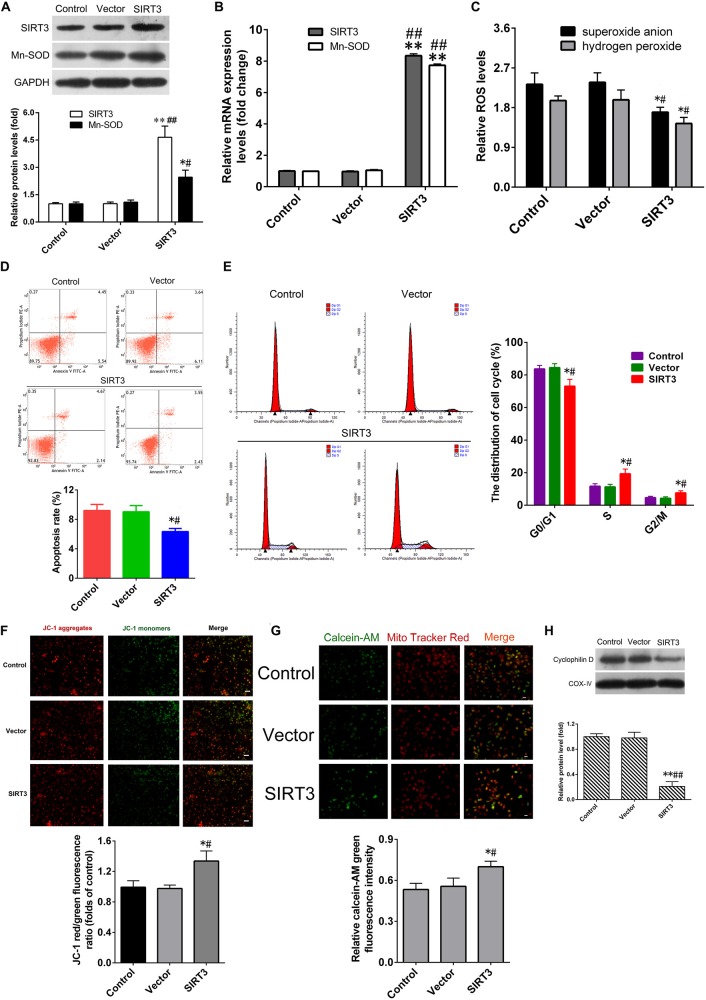
**SIRT3 overexpression alleviates cytotoxicity induced by activated microglia. (A)** Representative western blot (up panel) and quantification (down panel) of SIRT3, MnSOD expression in NSCs transfected with the recombinant adenoviral vector overexpressing SIRT3 (SIRT3 group) and empty vector (Vector group) for 48 h. **(B)** The mRNA expression of SIRT3, MnSOD in NSCs infected with empty vector or SIRT3 plasmid for 36 h. The reactive oxygen species (ROS) production **(C)**, cell apoptosis rate **(D)** and cell cycle distribution **(E)** in the Control group, Vector group and SIRT3 group were assessed by methods as showed in Figure 2, respectively. **(F)** Representative images (up panel) and quantification (down panel) of mitochondrial depolarization in NSCs transfected with SIRT3 plasmid for 48 h were assessed by JC-1 staining, *Scale bar* = 100 μm. **(G)** The mPTP opening in NSCs transfected with SIRT3 plasmid for 48 h was determined according to calcein-AM fluorescence intensity by flow cytometry. **(H)** Representative western blot (up panel) and densitometric quantification (down panel) of cyclophilin D protein expression in NSCs treated with empty vector or SIRT3 plasmid for 48 h. Results in **(A–C,F–H)** are expressed as fold change over that of Control group. Data are obtained in NSCs co-cultured with microglia challenged with 10 μM Aβ exposure. Data are presented as mean ± S.D. (*n* = 3). *P < 0.05, **P < 0.01 vs. the Control group. ^#^*P* < 0.05, ^##^*P* < 0.01 vs. the Vector group.

To confirm the protective role of SIRT3 overexpression in NSCs against microglia activation-induced cytotoxicity. After transfection with the SIRT3-expressing plasmid (SIRT3 group), the SIRT3 and MnSOD mRNA levels in NSCs were remarkably elevated by approximately 8-fold compared with that in untransfected cells (control group) or in cells transfected with the empty vector (vector group), the SIRT3 and MnSOD protein levels in NSCs of SIRT3 group were also higher than that in control or vector group (Figures 4A,B). SIRT3 overexpression significantly suppressed microglia activation-dependent ROS production (Figure 4C). The rate of cell apoptosis in SIRT3 group was lower than that in control or vector group (Figure 4D). SIRT3 overexpression also declined the proportion of G_0_/G_1_ phase cells significantly, while S and G_2_/M phase cells proportion increased dramatically (Figure 4E). SIRT3 overexpression suppressed microglia activation-dependent ΔΨm depolarization (Figure 4F) and mPTP opening (Figure 4G) in NSCs, consistent with decreased protein levels of CypD expression (Figure 4H). These data illustrated that SIRT3 overexpression in NSCs could ameliorate microglia activation-induced cell injury.

### Neuroprotective Effect of SIRT3 in NSCs Is Associated with Mitochondrial Apoptotic Pathway

**Figure 5:**
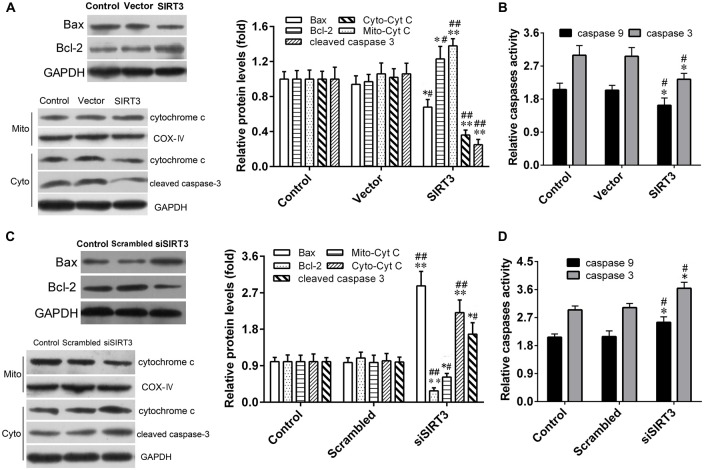
**SIRT3 in NSCs exerts neuroprotective effect through mitochondrial apoptotic pathway. (A)** Representative western blot (left panel) and quantification (right panel) of Bax, B-cell lymphoma 2 (Bcl-2), Mito-cytochrome C (Cyt C), Cyto-Cyt C and cleaved caspase-3 protein expression in NSCs treated with empty vector or SIRT3 plasmid for 48 h. **(B)** Caspase-3 and caspase-9 enzyme activities were detected by the spectrophotometric method in the Control group, Vector group and SIRT3 group. **(C)** Representative western blot (left panel) and quantification (right panel) of Bax, Bcl-2, Cyt C and cleaved caspase-3 protein expression in NSCs transfected with scrambled RNA or SIRT3 siRNA for 48 h. **(D)** Caspase-3 and caspase-9 enzyme activities were evaluated by the spectrophotometric method in the Control group, Scrambled group and siSIRT3 group. GAPDH and COX-IV were used as total cellular and mitochondrial protein markers, respectively, in **(A,C)**. Results are normalized to that of the Control group. Data are obtained in NSCs co-cultured with microglia challenged with 10 μM Aβ exposure. Data are presented as mean ± S.D. (*n* = 3). *P < 0.05, **P < 0.01 vs. the Control group. ^#^*P* < 0.05, ^##^*P* < 0.01 vs. the Vector or Scrambled group, respectively.

In order to explore the molecular mechanism underlying the neuroprotective effect of SIRT3 in NSCs against microglia activation-induced cytotoxicity. We next sought to investigate mitochondrial apoptotic pathway in oxidative stress-mediated central nervous diseases, the levels of several key molecules in the mitochondrial apoptotic pathway were measured by western blotting assay. The results depicted in Figure 5A revealed that protein levels of total cleaved caspase-3, Bax and cytoplasmic Cyt C decreased significantly in NSCs of SIRT3 group, which correlated with increased protein levels of total Bcl-2 and mitochondrial Cyt C compared to the control or vector group. On the contrary, compared to the control or scrambled group, SIRT3 knockdown in NSCs of siSIRT3 group made the protein levels of total cleaved caspase-3, Bax and cytoplasmic Cyt C up-regulated, the protein levels of total Bcl-2 and mitochondrial Cyt C down-regulated (Figure 5C).

In addition, the effect of SIRT3 on microglia activation-induced oxidative stress was also assessed by measuring the enzymatic activities of caspase-3 and caspase-9 in NSCs, which are pivotal pro-apoptotic factors. As shown in Figures 5B,D, SIRT3 overexpression effectively weakened the two enzymatic activities of caspase-3/9, while SIRT3 knockdown markedly enhanced them. These findings suggested that SIRT3 in NSCs alleviated microglia activation-induced oxidative stress injury through mitochondrial pathway, including the reduction of mitochondrial Cyt C released into cytoplasm, the decrease of Bax protein expression and caspase-3/9 enzymatic activity, along with the increase of Bcl-2 protein expression.

## Discussion

It was found in our study that Aβ aggravated NSC injury, accelerated cell apoptosis and slowed down cell cycle progression by activating microglia, which might be related with the oxidative stress inducers and inflammatory factors produced by activated microglia (Jiang et al., 2016). Decrease of mitochondrial membrane potential (ΔΨm) is an early event of cell apoptosis. mPTP is closely related with mitochondrial functions and cell survival, for example, increased mPTP opening promotes cell apoptosis (Du et al., 2014). It has been indicated in a study that high-level ROS induces mitochondrial dysfunction via mPTP open (Di Lisa et al., 2001). Mitochondrial heat shock protein 70 inhibits mPTP opening and, therefore, reduces Aβ-induced SH-SY5Y cell apoptosis (Qu et al., 2012; Du et al., 2014). Our finding showed that Aβ-activated microglia lowered mitochondrial membrane potential of NSCs, and enhanced the cell apoptosis rate by opening mPTP.

The results in our study demonstrated that Aβ down-regulated gene expression levels of SIRT3 and MnSOD in NSCs, which were further declined when microglia challenged with Aβ in co-culture system. Lipopolysaccharide (LPS) and inflammatory factor TNF-α significantly inhibit SIRT3 protein expression in human monocyte/macrophage, which presumably suggests that down-regulated expression of SIRT3 may be an outcome of inflammatory injury (Moschen et al., 2013). LPS promotes pericyte loss and inflammatory infiltration of cardiopulmonary tissue, which might be related with decreased SIRT3 protein expression (Zeng et al., 2016). H_2_O_2_-induced oxidative stress also results in reduced SIRT3 and MnSOD expression and risen cell apoptosis rate in human mesenchymal stem cells (Wang et al., 2014).

We found that overexpression of SIRT3 up-regulated MnSOD expression, decreased intracellular ROS level in NSC and relieved cell injury from oxidative stress. Reports in the literature indicate that calorie restriction reduces oxidative stress by SIRT3-mediated MnSOD activation (Qiu et al., 2010), and SIRT3 overexpression alleviates H_2_O_2_-induced oxidative stress injury in human mesenchymal stem cells (Wang et al., 2014). SIRT3 overexpression attenuates inflammatory damage by inhibiting LPS-induced pericyte loss and inflammatory infiltration of neutrophils/macrophages (Zeng et al., 2016), which further verifies the results of this study. Overexpression of SIRT3 can reverse lipotoxicity-mediated ROS accumulation and inflammation in the kidney, and decrease inflammatory cytokine monocyte chemoattractant protein-1 mRNA expression, whereas they are exacerbated by either overexpression of a dominant-negative form of SIRT3 (N87A) lacking deacetylase activity or knockdown of SIRT3 by siRNA transfection (Koyama et al., 2011). In our study, we reported that SIRT3 overexpression reduced microglia activation-induced mPTP open, ascended mitochondrial potential, and inhibited NSC apoptosis and protein expression of mitochondrial CypD, a member of mPTP. Cheng et al. (2016) reported that overexpression of SIRT3 ameliorated oxidative stress injury and ATP depletion in mitochondria, prevented mitochondrial swelling and mPTP open, thereby reduced neuron death. SIRT3 overexpression in C2C12 myoblast increases protein expression of MnSOD and PGC-1α, associated with elevated mitochondrial membrane potential (Padmaja Divya et al., 2015). Our previous studies also confirmed that overexpression of mitochondrial molecular chaperon HSP75 or inhibition of mPTP open by cyclosporine A exhibited some resistance to neurotoxicity caused by activated neuroglia cells (Wang et al., 2015; Chen et al., 2016). Suppression of CypD expression rescues Aβ-induced mitochondrial dysfunction and synaptic degeneration in neuron (Qu et al., 2012; Du et al., 2014; Valasani et al., 2016). We found that SIRT3 overexpression in NSCs inhibited CypD expression, decreased mPTP open, and relieved mitochondrial dysfunction caused by activated microglia. ROS production causes mitochondrial dysfunction through mPTP opening, in the process, NAD^+^ in mitochondria and cytoplasm is consumed excessively, which is necessary for SIRT3 to have its histone deacetylase activity (Di Lisa et al., 2001). In addition, it was also found in this study that SIRT3 overexpression reversed microglia activation-induced the increased proportion of G_0_/G_1_ phase cells and decreased proportion of S and G_2_/M phase cells significantly. SIRT3 relieves microglia activation-induced mitochondrial dysfunction in NSCs, however, the deeper molecular mechanism is still not so clear and needs further study in future.

Results of some reports supported our findings that SIRT3 overexpression reduced NSC apoptosis rate, conversely, SIRT3 deficiency increased the cell apoptosis rate. SIRT3 overexpression can protect dopaminergic neuron against neuronal death upon MPTP treatment. SIRT3 fights against cell injury by improving MnSOD activity and inhibiting expression of NF-κB and Bax (Zhang et al., 2016). Cheng et al. (2016) reported that SIRT3 knockdown endangered striatal and hippocampal neurons in mouse models of huntington’s disease and temporal lobe epilepsy.

We also found that SIRT3 overexpression down-regulated NSC’s pro-apoptotic factors Bax and cleaved caspase-3 expression, promoted anti-apoptosis factor Bcl-2 expression and decreased caspase-3/9 enzyme activity, while SIRT3 gene knockdown resulted in opposite outcomes. Ku70 is a new target of SIRT3, SIRT3 physically binds to Ku70 and deacetylates it, and this promotes interaction of Ku70 with the proapoptotic protein Bax. Thus, under stress conditions, increased expression of SIRT3 protects cardiomyocytes, in part by hindering the translocation of Bax to mitochondria (Sundaresan et al., 2008). Antioxidant caffeic acid can attenuate liver ischemia and reperfusion injury through regulating SIRT3, along with increased MnSOD expression and Bcl-2/Bax rate, and reduced cleaved caspase-3/9 protein expression (Mu et al., 2015). Rhamnetin protects H9c2 cardiomyoblasts against H_2_O_2_-induced apoptosis, its mechanisms of action include the enhanced expression of SIRT3, Bcl-2 and MnSOD, and the decreased protein expression of Bax and cleaved caspase-3 (Park et al., 2014). These findings can give some support to our results.

We also demonstrated that SIRT3 overexpression reduced the release of Cyt C from mitochondria into cytosol in NSCs, while SIRT3 deficiency enhanced the cytosolic accumulation of Cyt C, which was due to its release from the mitochondria. NAD^+^ and grape wine polyphenols prevent axonal apoptosis and act via mitochondrial SIRT3 activation, coincident with inhibited caspase-3 activation and the release of mitochondrial Cyt C in axons (Magnifico et al., 2013). Fucoidan suppresses mitochondrial dysfunction induced by traumatic brain injury in mice through elevated SIRT3 expression, which is evidenced by mitochondrial Cyt C release and collapse of mitochondrial membrane potential (Wang et al., 2016). These data can provide evidences for our findings.

**Figure 6:**
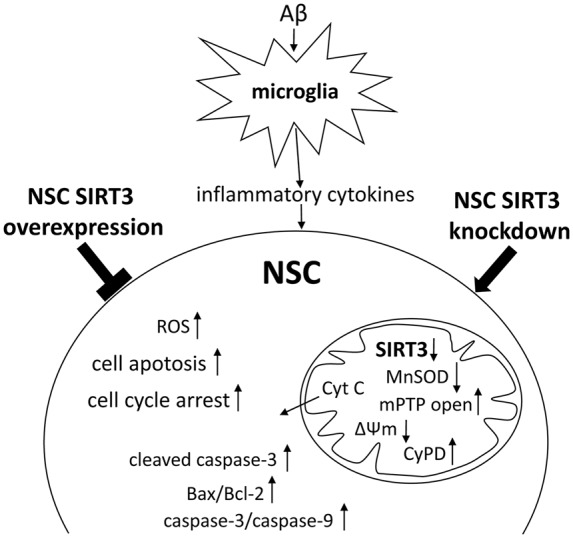
**Scheme summarizing the results.** Microglia activation by prolonged exposure of Aβ induced the accumulation of ROS and decreased SIRT3, MnSOD expression in NSCs, accompanied by cell cycle arrest, increased cell apoptosis and mPTP opening, and enhanced mitochondrial membrane potential (ΔΨm) depolarization. Furthermore, SIRT3 knockdown in NSCs accelerated the cell injury, whereas SIRT3 overexpression protected NSCs against activated microglia-induced cellular dysfunction through mitochondrial apoptosis pathway, including decreased mPTP opening and cyclophilin D (CypD) expression, inhibition of mitochondrial Cyt C release to cytoplasm, declined Bax/Bcl-2 ratio and caspase-3/9 enzymatic activity.

Taken together, our results revealed that oxidative stress produced by activated microglia may modulate NSC’s fate in the developing brain. SIRT3 in NSCs attenuated microglia activation-induced oxidative stress injury through mitochondrial apoptosis pathway, as summarized in Figure 6.

## Author Contributions

D-QJ designed and performed experiments and wrote the manuscript. YongW designed experiments and reviewed/edited the manuscript, YanW, M-XL and Y-JM read and corrected the manuscript. All authors approved the final manuscript.

## Conflict of Interest Statement

The authors declare that the research was conducted in the absence of any commercial or financial relationships that could be construed as a potential conflict of interest.
